# Pigmented Allergic Salute Sign in an Adult With Dark Skin: Clinical and Dermoscopic Features

**DOI:** 10.7759/cureus.102072

**Published:** 2026-01-22

**Authors:** Kawtar El Fid, Meryem Soughi, Sara Elloudi, Hanane Baybay, Fatima Zahra Mernissi

**Affiliations:** 1 Department of Dermatology, Hassan II University Hospital, Fez, MAR

**Keywords:** allergic rhinitis, allergic salute sign, dark skin, dermoscopy, hyperpigmentation

## Abstract

Post-inflammatory hyperpigmentation due to chronic mechanical friction is well recognized in individuals with darker skin types, yet the pigmented variant of the allergic salute sign remains rarely documented. We report a 40-year-old woman with a persistent transverse hyperpigmented band over the nasal dorsum, associated with long-standing allergic rhinitis and recurrent conjunctivitis. She described habitual upward rubbing of the nose due to intense pruritus. Clinical examination revealed a sharply demarcated dark linear band above the nasal tip. Dermoscopy demonstrated a homogeneous dark pseudo-network with prominent follicular openings, consistent with post-inflammatory hyperpigmentation in Fitzpatrick phototype VI skin. Awareness of this presentation is essential to differentiate it from lichen planus pigmentosus or discoid lupus erythematosus. Management focuses on controlling allergic rhinitis, minimizing mechanical friction, and cautious use of depigmenting therapies. This case highlights the pigmented allergic salute sign as a distinctive marker of chronic allergic disease in patients with dark skin.

## Introduction

The allergic salute is a habitual gesture observed in patients with allergic rhinitis, consisting of repetitive upward rubbing of the nose to relieve pruritus [1]. This mechanical friction classically results in transverse nasal creases or lichenification [2]; however, pigmentary sequelae are less frequently reported, particularly in adults and in individuals with dark skin phototypes [3]. In skin of color, friction-induced hyperpigmentation may present atypically and mimic other inflammatory or autoimmune dermatoses, leading to potential diagnostic confusion [4].

Pigmented variants of the allergic salute sign remain poorly documented in the literature, with very limited dermoscopic descriptions in adult patients. This case is reported to contribute to the clinical and dermoscopic characterization of this uncommon presentation and to raise awareness of frictional pigmentary disorders in patients with dark skin.

## Case presentation

A 40-year-old woman presented in March 2024 with a persistent hyperpigmented band on the nasal dorsum, which had gradually progressed over several years. Her medical history was notable for long-standing allergic rhinitis since childhood, associated with recurrent allergic conjunctivitis. The patient reported intense nasal pruritus and habitual upward rubbing of the nose, consistent with the allergic salute gesture.

**Figure 1:**
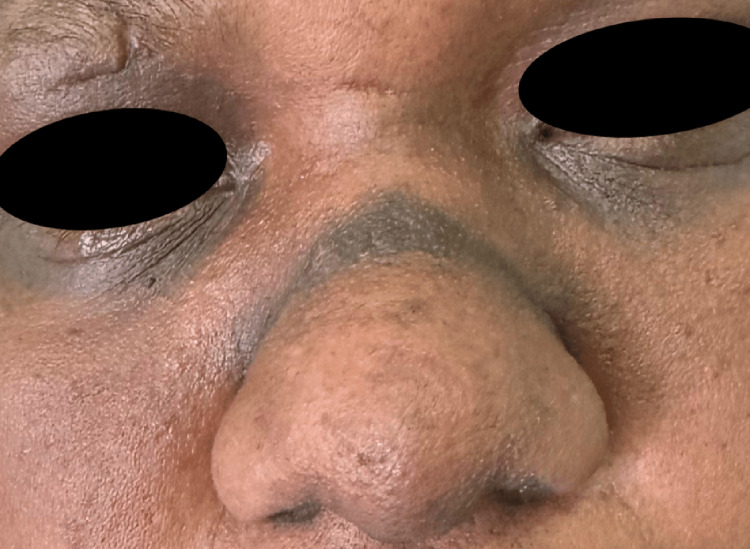
Close-up view of the nasal dorsum showing a sharply demarcated, hyperpigmented transverse band corresponding to the allergic salute crease.

**Figure 2:**
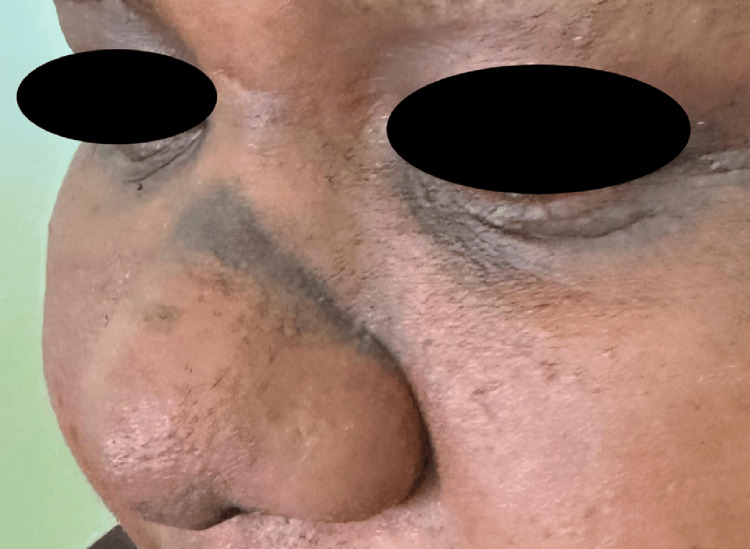
Lateral view of the nasal dorsum showing the same sharply demarcated, hyperpigmented transverse band at the site of the allergic salute crease.

Clinical examination revealed Fitzpatrick skin phototype VI and showed a sharply demarcated, dark-brown transverse macular plaque measuring approximately 3 cm in length and 0.8 cm in width, located above the nasal tip and precisely corresponding to the area subjected to repeated mechanical friction. Additional facial findings included marked periocular hyperpigmentation and bilateral infraorbital folds (double Dennie-Morgan lines), supporting an underlying atopic diathesis (Figures 1-2). Wood’s lamp examination was performed and showed partial accentuation of the pigmentation, consistent with a mixed-depth involvement (both epidermal and dermal components).

**Figure 3:**
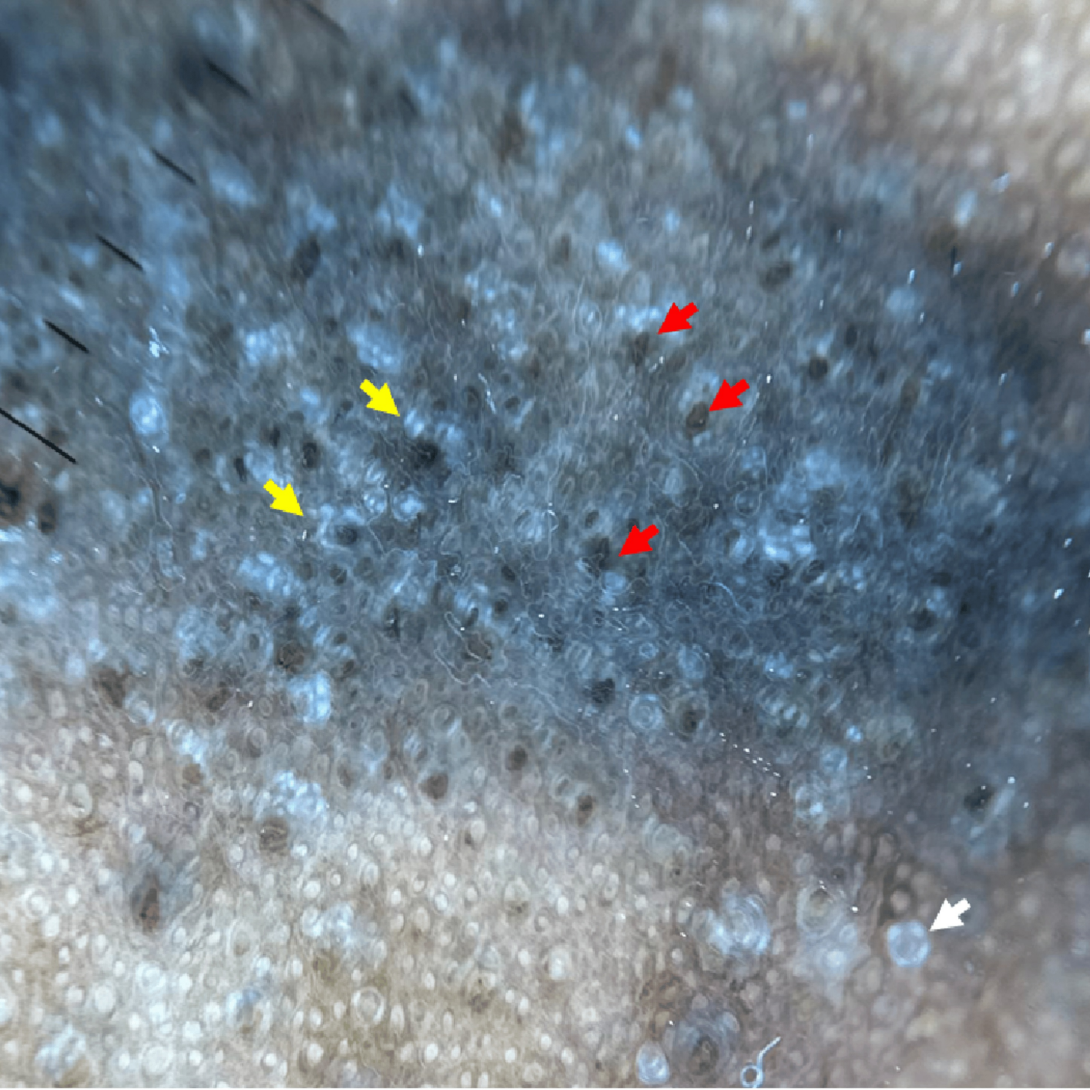
Dermoscopic view showing a homogeneous dark pseudo-network, follicular plugs (red arrow), crystalline structures (yellow arrow), and a rosette (white arrow).

Dermoscopy, performed in polarized mode, showed a homogeneous darkly pigmented pseudonetwork with a slightly grayish hue, and prominent blackish follicular openings diffusely and uniformly distributed throughout the lesion. Shiny whitish linear structures, corresponding to crystalline structures, were also observed (Figure 3). The dermoscopic pattern suggested a mixed-depth pigmentation, involving both epidermal and dermal components rather than purely epidermal pigmentation.

Given the patient’s atopic background, the chronic and progressive course of the lesion, the clear history of repeated friction, and the absence of clinical or dermoscopic features suggestive of discoid lupus erythematosus or lichen planus pigmentosus, a diagnosis of post-inflammatory hyperpigmentation induced by chronic friction related to the allergic salute gesture was made. A skin biopsy was not performed due to concerns regarding a potentially unfavorable cosmetic outcome.

Given the patient’s atopic background, the chronic and progressive course of the lesion, the clear history of repeated mechanical friction, and the absence of clinical or dermoscopic features suggestive of discoid lupus erythematosus or lichen planus pigmentosus, a diagnosis of post-inflammatory hyperpigmentation induced by chronic friction related to the allergic salute gesture was made. A skin biopsy was not performed due to concerns regarding a potentially unfavorable cosmetic outcome.

The patient was treated with topical hydroquinone 2% for three months, along with a facial moisturizer and daily sunscreen, with strict avoidance of mechanical friction. She was also referred to an otorhinolaryngologist for long-term management of allergic rhinitis; however, the patient was subsequently lost to follow-up.

## Discussion

The allergic salute gesture is common in patients with allergic rhinitis and may lead to chronic friction-induced skin changes [1]. While lichenification can occur across all skin phototypes, post-inflammatory hyperpigmentation tends to be more pronounced and persistent in individuals with darker skin [2-3]. An underlying allergic background, such as allergic conjunctivitis, may further predispose these patients to periorbital hyperpigmentation and affect other areas subjected to chronic scratching, as observed in our patient [5]. Given the high prevalence of allergic rhinitis worldwide, recognition of frictional pigmentary changes has important public health implications.

Despite its high prevalence, a distinctly pigmented presentation of the allergic salute sign in adults with dark skin has rarely been reported. To our knowledge, this case represents the first dermoscopic description of post-inflammatory hyperpigmentation resulting from chronic friction in this context. Dermoscopy revealed a homogeneous pigmented pseudonetwork with diffuse crystalline linear structures and prominent follicular plugs, along with other features commonly observed in frictional melanosis, such as perifollicular white coloration, focal structureless areas, white globules, and brown dots [4]. These dermoscopic findings help distinguish this entity from lichen planus pigmentosus, which shows dots, globules, and blotches in linear, reticular, or diffuse patterns with perifollicular accentuation, and discoid lupus erythematosus, characterized by whitish scales, arborizing vessels, follicular plugging, and starburst-like pigmentary changes [4,6].

Compared with the dermoscopic features of atopic dermatitis in skin of color, our case shows distinctive characteristics. Pediatric atopic lesions display patchy scales with early pigmentary changes and less conspicuous erythema, while adult atopic lesions exhibit more pronounced hyperpigmentation, *dirty* brown scales, and variable vascular patterns due to chronic inflammation [7-8]. By contrast, our patient showed a homogeneous dark pseudonetwork with prominent follicular plugs and shiny linear structures, without erythema or visible vessels, highlighting the persistent, uniform hyperpigmentation caused by chronic mechanical friction.

Beyond its diagnostic role, dermoscopy allows precise assessment of pigment distribution and depth, serving as a valuable non-invasive tool for therapeutic monitoring and follow-up, particularly in patients with skin of color [9]. Management relies on optimal control of allergic rhinitis, behavioral modification to reduce mechanical friction, and cautious use of depigmenting agents or laser therapies, carefully adapted to the skin of color to minimize adverse effects. Safer topical options, such as azelaic acid or topical calcineurin inhibitors, may be considered as alternatives to hydroquinone in selected cases [10].

## Conclusions

This case highlights the clinical and dermoscopic relevance of the pigmented allergic salute sign in patients with dark skin phototypes. Increased awareness of this entity may help prevent unnecessary investigations and misdiagnosis, allowing for appropriate management and improved aesthetic and symptomatic outcomes.

## References

[1] Greener M (2017). Allergic rhinitis: waving goodbye to the allergic salute. Independent Nurse.

[2] Nolan CF, Brelsford MA (2019). The metabolic salute: a unique presentation of transverse nasal acanthosis nigricans and allergic rhinitis in an obese pediatric patient. Pediatr Dermatol.

[3] Markiewicz E, Karaman-Jurukovska N, Mammone T, Idowu OC (2022). Post-Inflammatory hyperpigmentation in dark skin: molecular mechanism and skincare implications. Clin Cosmet Investig Dermatol.

[4] Sławińska M, Żółkiewicz J, Behera B (2023). Dermoscopy of inflammatory dermatoses (inflammoscopy) in skin of color - a systematic review by the International Dermoscopy Society “Imaging in Skin of Color” Task Force. Dermatol Pract Concept.

[5] Sheth PB, Shah HA, Dave JN (2014). Periorbital hyperpigmentation: a study of its prevalence, common causative factors and its association with personal habits and other disorders. Indian J Dermatol.

[6] Bhat YJ, Jha AK (2021). Dermatoscopy of inflammatory diseases in skin of color. Indian Dermatol Online J.

[7] Karampinis E, Toli O, Georgopoulou KE (2024). Exploring pediatric dermatology in skin of color: focus on dermoscopy. Life (Basel).

[8] Ankad B, Koti V (2020). Dermoscopic approach to inflammatory lesions in skin of color. Clinical Dermatol Rev.

[9] Errichetti E (2020). Dermoscopy in monitoring and predicting therapeutic response in general dermatology (non-tumoral dermatoses): an up-to-date overview. Dermatol Ther (Heidelb).

[10] Anvery N, Christensen RE, Dirr MA (2022). Management of post-inflammatory hyperpigmentation in skin of color: a short review. J Cosmet Dermatol.

